# Change in quality of life: a follow up study among patients with HIV infection with and without TB in Ethiopia

**DOI:** 10.1186/1471-2458-13-408

**Published:** 2013-04-29

**Authors:** Amare Deribew, Kebede Deribe, Ayalu A Reda, Markos Tesfaye, Yohannes Hailmichael, Todd Maja, Robert Colebunders

**Affiliations:** 1 Department of Epidemiology, Jimma University, Jimma, Ethiopia; 2 College of Public Health, Haromaya University, Alemaya, Ethiopia; 3 Department of psychiatry, Jimma University, Jimma, Ethiopia; 4 Department of Health Service management, Jimma University, Jimma, Ethiopia; 5 Department of Health Studies, UNISA, PO Box 392, Pretoria, South Africa; 6 Department of clinical Sciences, Institute of Tropical Medicine, Nationalestraat 155, Antwerp, 2000, Belgium; 7 Department of Epidemiology and Social Medicine, University of Antwerp, Campus Drie Eiken, Universiteitsplein 1, Antwerpen, 2610, Belgium

**Keywords:** TB/HIV co-infection, Quality of life, Antiretroviral treatment, Common mental disorders, Ethiopia

## Abstract

**Background:**

There is a dearth of literature on the impact of TB/HIV co-infection on quality of life (QoL). We conducted a study to assess the change in QoL over a 6-months period and its predictors among HIV-infected patients with and without TB in Ethiopia.

**Methods:**

465 HIV-infected patients without TB and 124 TB/HIV co-infected patients were enrolled in a prospective study in February, 2009. 455 (98%) HIV-infected and 97 (78%) TB/HIV co-infected patients were followed for 6 months. Data on QoL at baseline and 6^th^ month were collected by trained nurses through face to face interviews using the short Amharic version of the World Health Organization Quality of Life Instrument for HIV clients (WHOQOL HIV-Brief). Common Mental Disorder (CMD) was assessed using a validated version of the Kessler-10 scale. Multivariate analysis was conducted using generalized estimating equations (GEE) using STATA to assess change in QoL and its predictors.

**Results:**

There was a statistically significant improvement of the physical, psychological, social, environmental and spiritual QoL at the 6^th^ months follow up compared to the baseline for both groups of patients (P < 0.0001). The change in QoL in all dimension were more marked for TB/HIV co-infected patients compared to HIV-infected patients without TB.

A severe form of CMD was strongly associated with poorer physical QoL among TB/HIV co-infected individuals (β = −2.84; P = 0.000) and HIV clients without TB (β = −2.34; P = 0.000).

**Conclusion:**

This study reveals that ART and anti-TB treatment significantly improve the QoL particularly among TB/HIV co-infected patients. We recommend that the ministry of health in collaboration with partners shall integrate mental health services into the TB/HIV programs and train health care providers to timely identify and treat CMD to improve QoL.

## Background

The advent of anti-retroviral therapy (ART) and its widespread availability in many settings has reduced the mortality rate among people living with HIV/AIDS (PLHA) [1]. As longevity of PLHA improves as a result of ART, improvement of quality of life (QoL) of these patients has become an important issue for researchers and policy makers [2]. According to the World Health Organization (WHO), QoL is defined as an individuals’ perception of their position in life in the context of the culture and value systems in which they live and in relation to their goals, expectations, standards and concerns [2]. This definition considers individuals’ satisfaction on their physical, psychological, social relationships, environment, and spiritual aspects of their life [3].

In addition to its biological and physical burden, HIV/AIDS is associated with many social consequences such as stigma and discrimination which have negative impacts on QoL [4-6]. QoL is also affected by several clinical and socio-demographic factors. In a prospective cohort study conducted in 2003 and 2004 among 947 HIV-infected adults initiating highly active antiretroviral therapy in Uganda, the overall score of QoL significantly increased from the baseline and most of the gains were achieved by the third month of therapy. While several clinical, psychosocial and socio-demographic factors predicted QOL at ART initiation, financial dependence on others was the only remaining predictor after controlling for time on ART [7]. Another cohort study among men in USA showed that higher family support and CD4 lymphocyte counts at baseline were predictive of improved changes in physical and social functioning over time, and higher depressive symptoms at baseline were predictor of diminished role functioning, emotional well-being, and general health perception [8]. Whereas a prospective cohort study conducted among 1053 patients in France revealed that baseline CD4 lymphocyte count, time since HIV diagnosis, undetectable viral load, and lower number of self-reported symptoms were predictors of QoL [9].

Other predictors of QoL include poor social support [10], depression [6,10,11], unemployment [12] or financial dependence on others [6,7], older age [13] and being female [14].

Many studies have documented significant improvements in QoL during ART [7,13,15]. There is however a dearth of literature on the impact of tuberculosis (TB)/HIV co-infection on QoL. From February to April 2009, we conducted a QoL survey among HIV infected patients with and without TB in three hospitals in Ethiopia [6]. This study showed that TB/HIV co-infected patients had poorer QoL life than HIV infected patients without TB. In this paper, we describe the change in QoL of the same patients after 6-months of treatment.

## Methods

### Study settings and population

From February to April 2009, 465 HIV-infected patients without TB and 124 TB/HIV co-infected patients who were taking ART in three hospitals in Ethiopia were enrolled in a prospective study. The patient selection process was described in our previous paper [6]. In brief, at Jimma, Nekemte and Adama hospitals, for each TB/HIV co-infected patient, 3 HIV-infected patients without TB were selected using a simple random sampling technique. All TB/HIV co-infected patients were in the intensive phase of anti-TB treatment. Exclusion criteria for both groups included age less than 15 years, the presence of an opportunistic infection or a known chronic illness like diabetes mellitus and hypertension. Patients were followed up monthly.

### Data collection procedures and follow up

Diagnoses of TB and HIV were based on national guidelines [16]. Smear microscopy was the major diagnostic tool for pulmonary TB. TB lymphadenitis was diagnosed based on clinical parameters and cytological examination of an aspirate obtained by fine needle aspiration. Patients with HIV infection were considered not to have TB if they did not present with any of the TB clinical symptoms [16]. During each clinic visit, patients were thoroughly assessed by trained nurses for drug side effects, general health status and presence of symptoms of opportunistic infection including TB. CD4 lymphocyte count and WHO clinical staging were extracted from the patients’ record at baseline and at the 6^th^ months of follow up. QoL was measured at baseline and after 6 months of follow up through face to face interviews using the short Amharic version of the World Health Organization QoL Instrument for HIV infected patients (WHOQOL HIV-Brief) [17]. This QoL instrument has been described in our previous article [6]. In brief, it consisted of 31 Likert scale questions in 6 domains of QoL: Physical health (4 items); psychological wellbeing (5 items); social relationship (4 items); environmental health (8 items); level of independence (4 items) and spiritual health (4 items). There were two questions about general QoL and perceived general health.

Common Mental Disorder (CMD) was measured using the Kessler 10 scales [18]. This instrument has 10 questions each asking the respondent how often they experienced symptoms during the previous 30 days and containing 5-point Likert scales (1 = never, 2 = a small part of the time, 3 = some of the time, 4 = most of the times, 5 = all of the time). The Kessler-10 scale was validated in Ethiopia and used extensively [19,20].

### Data analysis

Data were analyzed using the SPSS version 16.0 and STATA® version 11 software. Domain scores in the WHOQOL-HIV-Brief were scaled in positive direction with higher score denoting good quality of life. Negative questions like pain and discomfort were recorded so that higher scores reflected better QoL. Mean scores of items within each domain were used to calculate the domain score. Mean scores were then multiplied by 4 in order to make domain scores comparable with the scores used in the World Health Organization QoL (WHOQOL-100). We used t-test and F-test to compare means between groups.

QoL was treated as continuous variable. CMD was categorized as normal (score <20), moderate (score 20–24), severe (score 25–29) and very severe (score above 30) as proposed previously [18,21]. Paired T-test was used to compare repeated measurements of QoL at baseline and at 6 month. Multivariate analysis was conducted using generalized estimating equations (GEE) using the Gaussian family and the identity link function. In this model, the correlations between the baseline and 6-month measurements were taken into account. Variables with significant correlation were removed since the model automatically detects correlations. A P-value of less or equal to 0.05 was taken as the cut-off value for statistical significance.

#### Ethical clearance

Ethical clearance was obtained from the Jimma University ethical review board. Written informed consent was obtained from the study participants. To ensure confidentiality, the data were anonymised before they were analyzed.

## Results

After 6 months, 455 (98%) of the 465 HIV-infected patients and 97 (78%) of the 124 TB/HIV co infected patients were still in follow up. Thirty seven (6.3%) were lost to follow-up. There were no significant difference in baseline CD4 lymphocyte count between patients who completed the study and those lost to follow up, nonetheless most of the lost to follow-up were in WHO stage 3 and 4(P = 0.002).

Over the 6 months period, 5/455(1.1%) patients with HIV infection developed pulmonary TB, 7 (7.2%) of TB/HIV co-infected patients and 10 (1.8%) of all patients missed their anti-TB treatment and ART at least once. Twenty five percent of TB/HIV co-infected patients had their CD4 lymphocyte measured both at baseline and after 6 months, compared to 35% of HIV infected patients without TB (Table 1).

**Table 1:** Socio-demographic and clinical characteristics of TB/HIV co-infected and HIV-infected patients without TB

**Variables**	**TB/HIV co-infected patients (n = 97)**	**HIV-infected patients without TB (n = 455)**	**P-Value**
Age in years, Mean (SD)	34.5 (9.5)	33.4 (8.1)	0.2
Sex			
Male	43 (44.3%)	185 (40.6%)	0.5
Female	54 (55.7%)	270 (59.4%)	
Employment			
Unemployed	7 (7.2%)	52 (11.4%)	0.2
Employed	90 (92.8%)	403 (88.6%)	
Have income source			
Yes	69 (71.1%)	316 (69.5%)	0.7
No	28 (28.9%)	139 (30.5%)	
Have social support			
Yes	11(11.3%)	63 (13.8%)	0.5
No	86 (87.7%)	392 (86.2%)	
WHO staging			
Stage I	-	71(15.6%)	0.000
Stage II	-	64 (14.1%)	
Stage III	79 (81.4)	216 (47.5%)	
Stage IV	18 (18.6%)	65 (14.2%)	
Mean CD4 lymphocyte count, (median)	392.6 (375.0) (n = 24)	383.7 (358.0) (n = 160)	0.8
Missed at least one dose of anti-TB treatment	7 (7.2%)	-	-
Missed at least one dose of ART	3(3.1)	7 (1.5%)	0.535
Lost to follow-up	27(21%)	10 (2.2%)	0.000

### Change in quality of life

In all patients, after 6 months of treatment there was a significant improvement of QoL in all its dimensions (physical, psychological, social relationships, environmental, spiritual and level of dependence) (Table 2 and 3).

**Table 2:** Change in QoL among TB/HIV co-infected patients after 6 months of treatment

**QOL domain**	**Mean (SD) at baseline**	**Mean (SD) after 6 months**	**Mean difference (95% CI)**	**P-value**
Physical	13.3 (4.4)	17.9 (2.5)	5.1 (4.2 – 6.0)	0.000
Psychological	15.0 (3.2)	17.9 (1.8)	3.3 (2.6 – 4.0)	0.000
Level of independence	11.7 (3.7)	17.0 (2.4)	5.7 (4.9 – 6.6)	0.000
Social relationships	12.2 (3.7)	14.5 (2.5)	2.6 (1.7 – 3.5)	0.000
Environmental health	11.6 (3.1)	14.2 (2.7)	2.8 (2.1 – 3.6)	0.000
Spirituality health	16.5 (4.0)	19.0 (1.7)	3.1 (2.3 – 4.0)	0.000

**Table 3:** Change in QoL among HIV infected patients without TB after 6 months of treatment

**QOL domain**	**Mean (SD) at baseline**	**Mean (SD) after 6 months**	**Mean difference (95% CI)**	**P-value**
Physical	16.8 (2.8)	17.5 (2.5)	0.7 (0.4 – 1.1)	0.000
Psychological	16.2 (2.5)	17.4 (2.2)	1.2 (0.9 – 1.5)	0.000
Level of independence	15.0 (2.8)	16.6 (2.6)	1.6 (1.3 – 2.0)	0.000
Social relationships	13.6 (2.9)	14.3 (2.8)	0.6 (0.2 – 1.0)	0.002
Environmental health	12.4 (2.7)	13.7 (2.7)	1.2 (0.9 – 1.6)	0.000
Spiritual health	17.9 (2.9)	18.5 (2.3)	0.7 (0.4 – 1.0)	0.000

The change in QoL in all its dimension was more pronounced for TB/HIV co-infected patients compared to HIV infected patients without TB. For instance, there was a 4.4 unit difference between TB/HIV co-infected and HIV infected patients without TB in the physical dimension of QoL (Table 4).

**Table 4:** Comparison of QoL among TB/HIV co-infected and HIV-infected patients without TB

**QOL domain**	**Mean difference for TB/HIV co-infected**	**Mean Difference for HIV-infected without TB**	**Mean difference of differences (95% CI)**	**P-value**
Physical	5.1 (4.2 – 6.0)	0.7 (0.4 – 1.1)	4.4 (3.4 - 5.4)	0.000
Psychological	3.3 (2.6 – 4.0)	1.2 (0.9 – 1.5)	2.1 (1.4 - 2.9)	0.000
Level of independence	5.7 (4.9 – 6.6)	1.6 (1.3 – 2.0)	4.1 (3.2 - 5.0)	0.000
Social relationships	2.6 (1.7 – 3.5)	0.6 (0.2 – 1.0)	1.2 (1.1 - 2.9)	0.000
Environmental health	2.8 (2.1 – 3.6)	1.2 (0.9 – 1.6)	1.6 (0.8 - 2.4)	0.000
Spiritual health	3.1 (2.3 – 4.0)	0.7 (0.4 – 1.0)	2.4 (1.5 - 3.3)	0.000

### Predictors of change in QoL

Although not statistically significant, lack of social support, absence of a source of income and poor adherence to ART had a negative effect on the physical dimension of QoL of all patients. A severe form of CMD was strongly associated with poorer physical QoL among TB/HIV co-infected individuals (β = −2.84; P = 0.000) and HIV infected patients without TB (β = −2.34; P = 0.000) (Table 5).

**Table 5:** Predictors of physical QoL among TB/HIV co-infected and HIV-infected patients without TB

	**TB/HIV co-infected**		**HIV-infected without TB**	
**Variables**	**β (SE)**	**P-value**	**β (SE)**	**P-value**
**Sex**				
Male	1.00		1.00	
Female	−0.26 (0.4)	0.5	0.07	0.7
**Mean CD4 count**				
<50	1.00		1.00	
50-100	0.98 (0.8)	0.2	0.13 (0.3)	0.7
101-200	0.28 (0.8)	0.7	0.17 (0.28)	0.5
>200	0.20 (0.8)	0.8	0.19 (0.29)	0.5
**WHO staging**				
I	NA		1.00	
II	NA		−0.11 (0.3)	0.7
III	1.00		−0.28 (0.3)	0.3
VI	−0.91 (0.6)	0.1	0.05 (0.3)	0.9
**Adherence to ART**				
Yes	1.00		1.00	
No	−0.61 (1.2)	0.6	−0.31 (0.5)	0.5
**Adherence to anti-TB**				
Yes	1.00		NA	
No	0.27 (1.4)	0.8		
**Social support**				
Yes	1.00		1.00	
No	−0.27 (0.5)	0.6	0.11 (0.3)	0.7
**Source of income**				
Yes	1.00		1.00	
No	−0.49 (0.6)	0.4	−0.35 (0.2)	0.07
**CMD**				
Normal	1.00		1.00	
Moderate	−0.21 (0.7)	0.8	−0.69 (0.2)	0.001
High	−0.88 (0.6)	0.2	−1.37 (0.2)	0.000
Very high	−2.48 (0.6)	0.000	−2.34 (0.3)	0.000
**Employment**				
Yes	1.00		1.00	
No	0.42 (0.7)	0.6	−0.17 (0.3)	0.6

*NA = not applicable.

Though not statistically significant, lack of social support had a negative effect on psychological QoL of TB/HIV co-infected patients (β = −0.28, P = 0.4) and HIV infected patients without TB (β = −0.44, P = 0.2). A severe form of CMD was strongly associated with poorer psychological QoL in both groups of patient (Table 6).

**Table 6:** Predictors of psychological QoL among TB/HIV co-infected and HIV-infected patients without TB

	**TB/HIV co-infected**		**HIV-infected patients without TB**	
**Variables**	**β (SE)**	**P-value**	**β (SE)**	**P-value**
**Sex**				
Male	1.00		1.00	
Female	−0.69 (0.3)	0.03	−0.22 (0.2)	0.1
**Mean CD4 count**				
<50	1.00		1.00	
50-100	1.15 (0.6)	0.06	−0.16 (0.3)	0.6
101-200	0.70 (0.6)	0.2	−0.12 (0.2)	0.6
>200	0.53 (0.6)	0.3	−0.01 (0.2)	0.969
**WHO staging**				
I			1.00	
II			−0.36 (0.3)	0.2
III	1.00		−0.10 (0.3)	0.7
VI	−0.42 (0.4)	0.3	−0.04 (0.3)	0.9
**Adherence to ART**				
Yes	1.00		1.00	
No	0.06 (0.8)	0.9	0.40 (0.5)	0.4
**Adherence to anti-TB**				
Yes	1.00		NA	
No	0.43 (1.0)	0.7		
**Social support**				
Yes	1.00		1.00	
No	−0.28 (0.4)	0.5	−0.44 (0.2)	0.06
**Source of income**				
Yes	1.00		1.00	
No	−0.06 (0.4)	0.9	−0.56 (0.2)	0.001
**CMD**				
Normal	1.00		1.00	
Moderate	−0.87 (0.5)	0.08	−0.51 (0.2)	0.010
High	−0.76 (0.4)	0.09	−1.71 (0.2)	0.000
Very high	−2.29 (0.4)	0.000	−2.04 (0.3)	0.000
**Employment**				
yes	1.00		1.00	
No	0.04 (0.5)	0.9	−0.40	0.1

## Discussion

This study shows that all the dimensions of QoL have significantly improved after 6 months of treatment in both patient groups. However, the improvement in QoL was more pronounced for TB/HIV co-infected patients. The significant improvement of the physical quality of life of TB/HIV co-infected patients could be explained by the relief of TB symptoms during anti-TB treatment [22]. Some studies have documented that the major gain in QoL among patients with HIV infection occurs in the first three months of initiating of ART [8,23].

In our study, income and employment did not influence significantly QoL but this may be because of the small sample size of the study and most study participants had a similar socio-economic background. Indeed, other studies have shown that socio-economic status and social support are very important predictors of QoL of persons with HIV infection [12,16]. Gender also did not predict QoL in our study in contrast with some other studies that reported lower QoL among women [14,24,25]. This could be due to the presence of some income generating schemes by Civil Society Organizations targeting only HIV infected women in Ethiopia.

In contrast with other studies, we were unable to demonstrate that a lower CD4 lymphocyte count did not predicted a worse QoL [17]. This could be because of the small sample size of the study and secondly, all patients received adequate treatment with good adherence rate.

In our study, the presence of CMD was a main predictor of QoL after controlling other confounding variables. This suggests that screening and treatment of CMDs could be an important strategy towards improving the QoL of patients with TB and/or HIV in Ethiopia.

Our study has several limitations. First, the lost to follow up due to unknown reasons and missing data on CD4 count, particularly among TB/HIV co-infected patients, may have obscured the effect of CD4 lymphocyte count and WHO clinical staging on quality of life. Second, the cause-effect relationship between CMD and QoL could not be established. Third, we did not gather comprehensive information regarding income and occupation which might have impact on QoL.

## Conclusion

Our study shows that ART and anti-TB treatment improves all dimensions of QoL. CMD is a major predictor of poor QoL. We recommend that the ministry of health in collaboration with partners shall integrate mental health services into the TB/HIV programs and train health care providers to timely identify and treat CMD to improve QoL.

## Competing interests

The authors declare that they have no competing interests.

## Authors’ contributions

AD conceived the study and was involved in the design, analysis and report writing. MT participated in the design and reviewed the article. AR analyzed the data and reviewed the article. YH was involved in report writing and reviewing. KD was involved in analysis and write up. TM has reviewed the article extensively. RC participated in the design and critically reviewed the article. All authors read and approved the final manuscript.

## Pre-publication history

The pre-publication history for this paper can be accessed here:

http://www.biomedcentral.com/1471-2458/13/408/prepub
